# Antioxidative and Cytoprotective Efficacy of Ethanolic Extracted Cranberry Pomace against *Salmonella* Enteritidis Infection in Chicken Liver Cells

**DOI:** 10.3390/antiox12020460

**Published:** 2023-02-11

**Authors:** Nada Ahmed, Mohamed El-Fateh, Magdy S. Amer, Reham A. El-Shafei, Muhammad Bilal, Moussa S. Diarra, Xin Zhao

**Affiliations:** 1 Department of Animal Science, McGill University, Sainte-Anne-de-Bellevue, Montreal, QC H9X 3V9, Canada; 2 Department of Pharmacology, Faculty of Veterinary Medicine, Mansoura University, El-Dakhelia, Mansoura 35516, Egypt; 3 Department of Hygiene and Zoonoses, Faculty of Veterinary Medicine, Mansoura University, El-Dakhelia, Mansoura 35516, Egypt; 4 Guelph Research and Development Center, Agriculture and Agri-Food Canada, Guelph, ON N1G 5C9, Canada

**Keywords:** cranberry pomace extract, antioxidant regulatory genes, cytoprotecting, *S. Enteritidis*, anti-infection

## Abstract

*Salmonella enterica* serovar Enteritidis is a globally significant zoonotic foodborne pathogen. Chicken liver is a vital organ that has been recently implicated in several reported human salmonellosis outbreaks in the U.S. One promising strategy for reducing Salmonella in chickens could be through supplementation with natural antimicrobial additives. Ethanolic extracted cranberry pomace (CPOH) is an excellent source of bioactive polyphenolic compounds with antioxidant and antimicrobial activities. However, the protective effect of CPOH against *S. Enteritidis*-induced chicken hepatic cell damage remains unclear. In this study, we used a chicken hepatoma cell (LMH) infection model to investigate the protective effects and potential mechanisms of CPOH. CPOH increased the viability of *S. Enteritidis*-infected LMH cells. Furthermore, CPOH reduced the adhesion and invasion of *S. Enteritidis* to LMH cells. CPOH downregulated the expression of Rho GTPase genes that are essential for Salmonella’s entry into LMH cells. Additionally, the expression of antioxidant regulatory genes, such as Nrf2, HO-1, Txn, and Gclc, was increased. Our data show that CPOH effectively protected LMH cells from cell damage through the inhibition of *S. Enteritidis* adhesion and invasion, as well as the induction of the expression of master antioxidant genes. These findings offer opportunities to develop sustainable, safe, and economic strategies to reduce the colonization and pathogenesis of *Salmonella*.

## 1. Introduction

Poultry is a major livestock species in animal farming and poultry meat is the most widely consumed meat. Nevertheless, consumers are still concerned with meat quality, safety, and health benefits [1]. The public desires products from poultry raised without antibiotics, which has led the industry to restrict or ban the use of prophylactics of antibiotics while looking for potent antibiotic alternatives in production [2].

Chicken liver is one of the most dynamic organs, performing a various array of functions. Although the liver has so many essential functions for a bird’s health, a “physiologically normal” liver is rarely seen in field conditions. The liver injury is frequently accompanied with intestinal barrier impairment as the two organs affect each other through the gut–liver axis [3]. In addition, any damage in the gut epithelium tight junctions may be the main pathological mechanism underlying bacterial translocation from the gut to the liver [4]. Hence, chicken liver has been recognized as an important source for foodborne infections [5].

Non-typhoidal *Salmonella enterica* serovars continue to be an important food safety issue worldwide. According to CDC reports, *Salmonella* accounts for 153 million cases of gastroenteritis and 57,000 deaths globally each year [6]. The infected poultry products (meat and egg) are some of the main contributors to the worldwide salmonellosis burden. In particular, chicken liver has been recently added as an important foodborne illness vehicle due to the increasing number of salmonellosis outbreaks in the U.S. [7]. The ability of *Salmonella* to adhere to and invade into mammalian cells is a crucial step to initiate infection. In chickens, *Salmonella* can invade both phagocytic and non-phagocytic cells to disseminate through the bloodstream, causing systemic infection and the invasion of various internal organs such as the liver, spleen, ovaries, and oviduct [8]. Invasion of *Salmonella* into non-phagocytic cells is mediated by the activation of the host Rho GTPases *Cdc-42, Rac-1,* and *RhoG*, leading to actin cytoskeletal reorganization, membrane ruffling, and bacterial uptake through macropinocytosis [9]. Once inside the cell, the survival of *Salmonella* and outcome of infection are dependent on various bacterial and host factors. Nuclear factor erythroid-derived 2-related factor 2 (Nrf2) provides the main cytoprotective defense system in the host cell and is known as a master regulator of tissue damage and disease tolerance to infection via the coordinated regulation of the glutathione (GSH) and thioredoxin (Txn) antioxidant system, heme and iron metabolism, ROS, and xenobiotic detoxification to avoid DNA damage and cell death [10]. *Salmonella* cytotoxic effector proteins could attenuate the antioxidant activities of Nrf2 [11] and Txn, which in turn can induce human epithelial cell injury and death [12]. However, there has been no study concerning the cytotoxic effect of *S. Enteritidis* on the antioxidant system in chicken hepatic epithelial cells.

The control of *Salmonella* in poultry production is complex and requires concerted efforts, especially due to the development of antibiotic resistance and the high strain specificity of bacteriophages [13]. Therefore, there is an urgent need to control *S. Enteritidis* in the poultry industry to effectively reduce the threat of salmonellosis in humans. In recent years, the use of phytochemicals (natural compounds from plant extracts) has gained attention. Dietary phytochemicals are effective, non-resistance-forming, renewable, economical, and environment friendly [14]. Among them, American cranberry (*Vaccinium macrocarpon*) pomace and extracts are good sources of bioactive polyphenolic compounds having a wide range of biological activities, including antioxidant, antimicrobial, and anti-inflammatory activities [15,16]. In the context of the circular economy, the use of cranberry residue (pomace) is an intriguing strategy for reusing food industry waste, as it contains various nutraceutical molecules such as phenolic compounds with health benefits [17]. Surprisingly, acidic CPOH extracts possess antimicrobial activities against *Salmonella enterica* serovars from chickens with a minimum inhibitory concentration (MIC) of 8 mg/mL [18]. Moreover, the dietary supplementation of a polyphenol-rich cranberry extract exhibited noteworthy hepatoprotective and anti-inflammatory efficacy in high-fat-fed obese mice [19]. However, few studies have examined the impact of CPOH or its extracts on host cells during their interaction with pathogenic bacteria. Accordingly, the objective of this work was to determine (1) whether polyphenol-rich CPOH could protect chicken LMH cells following infection by *S. Enteritidis* and (2) how polyphenol-rich CPOH could protect chicken LMH cells following infection by *S. Enteritidis*. This is the first study to examine the role of cranberry by-products in *S. Enteritidis*-invaded poultry liver cells.

## 2. Materials and Methods

### 2.1. Cell Culture and Treatment

The chicken LMH cells (ATCC, CRL-2117) were used as the hepatic epithelial cell model for this study. LMH cells were cultured in Waymouth’s medium (Gibco, Thermo Fisher Scientific, Inc., Waltham, MA, USA) with 10% fetal bovine serum (FBS) (Gibco, Thermo Fisher Scientific, Inc., USA), 100 U/mL of penicillin, and 100 μg/mL of streptomycin (Gibco, Thermo Fisher Scientific, Inc., USA). All flasks and plates were precoated with the attachment factor protein (1×) (Gibco, Thermo Fisher Scientific, Inc., USA) containing gelatin for cell adherence to surfaces. The cells were incubated in a humidified environment of 5% CO_2_ at 37 °C. In this study, the cells received 8 treatments as follows: control group (Con), LMH cells treated with 1 mg/mL CPOH (CPOH1), LMH cells treated with 2 mg/mL CPOH (CPOH2), LMH cells treated with 4 mg/mL CPOH (CPOH4), LMH cells infected with *S. Enteritidis* (S), LMH cells simultaneously infected with *S. Enteritidis* and treated with 1 mg/mL CPOH (S + CPOH1), LMH cells simultaneously infected with *S. Enteritidis* and treated with 2 mg/mL CPOH (S + CPOH2), and LMH cells simultaneously infected with *S. Enteritidis* and treated with 4 mg/mL CHOH (S + CPOH4).

### 2.2. Culture of Salmonella enterica Serovar Enteritidis (S. Enteritidis)

*Salmonella enterica* serovar Enteritidis 43353 was used in this study. It has been used as a strain for the development of *S. enterica*-specific infections [20,21]. The *S. Enteritidis* strain 43353 from frozen stocks at −20 °C was inoculated in 5 mL Luria–Bertani (LB) broth (Difco, Sparks, MD, USA) and grown overnight at 37 °C with vigorous shaking (180 rpm). The overnight cultured bacteria were centrifuged at 8000 rpm for 10 min to pellet cells and re-suspended in phosphate-buffered saline (PBS, pH 7.0) (Gibco, Thermo Fisher Scientific, Inc., USA) to the desired density, which was confirmed by colony counting on the LB agar.

### 2.3. Ethanolic Extracted Cranberry Pomace

The ethanolic extract of cranberry pomace (CPOH) was prepared from organic cranberry (*V*. *macrocarpon*) pomace as previously described [16]. It was supplied by Dr. Moussa S. Diarra (Guelph Research and Development Center, Agriculture and Agri-Food Canada, Guelph, ON, Canada). Briefly, phenolic-rich compounds were extracted with 80% ethanol from the cranberry pomace. After extraction, the ethanol was removed by evaporation and the remaining extracts were freeze-dried at −30 °C for 11 days to generate an acidic pomace extract (CPOH) and stored at −20 °C.

The physical properties and chemical composition of the CPOH used in the present study were previously determined and reported by Ross et al. [16]. Briefly, the CPOH used in the study contained 24.87 ± 0.66 mg of gallic acid eq./g total phenolic content, with a pH of 2.74 ± 0.02 and 77.77 ± 0.52% sugar. Prior to each assay, the freeze-dried CPOH was weighed and dissolved in the Waymouth’s medium to obtain fresh stock solutions measuring 50 mg/mL. Then, it was sterilized by passage through a sterile 0.2 µm syringe filter. To neutralize the acidity of the CPOH, the stock of the extract was adjusted at a pH of 7.0 using a presterilized 4 M NaOH solution [22]. From this neutralized stock solution, the treatment doses of 1, 2, and 4 mg/mL (CPOH1, CPOH2, and CPOH4) were prepared in the Waymouth’s medium containing 10% FBS.

### 2.4. Determination of Minimum Inhibitory Concentrations (MICs) of Acidic and Neutral Cranberry Pomaces (CPOHs) against S. Enteritidis

The MICs were determined using a broth micro-dilution method according to the Clinical Laboratory Standard Institute’s (CLSI’s) guidelines [23]. The stock solutions (128 mg/mL) of sterile CPOH (either acidic or neutralized) were diluted by two-fold serial dilutions using 96-well plates in a concentration ranging from 0 to 64 mg/mL, followed by the addition of 100 μL of overnight cultures of *S. Enteritidis* in each well at a final concentration of 10^5^ CFU/mL, resulting in a final well volume of 200 μL. The plates were loaded in a microplate reader (Spark, Tecan, Switzerland) after 24 h of incubation at 37 °C to record the optical densities at 600 nm (OD600 nm). Media without inoculum but with the studied concentrations of the tested products were included as blanks. The OD with blanks was subtracted from the OD of inoculated wells containing equivalent concentrations of studied products. The MIC was determined as the minimum concentration of CPOH at which no increase in optical density was observed over 24 h. Ceftiofur (Sigma Aldrich) was used as a control antibiotic.

The minimal bactericidal concentration (MBC) was determined using a spot assay after 24 h of growth, as the concentration of tested products needed to kill at least 99.9% of the initial inoculums as determined by plating a sample from each well with no visible growth on the LB agar after an overnight incubation at 37 °C.

### 2.5. Cell Viability Assay

The cell viability was determined using a Cell Counting Kit-8 (WST-8/CCK8) (Abcam, Cambridge, MA, USA). In brief, the LMH cells were first seeded on 96-well plates (Corning, Inc., Corning, NY, USA) (1 × 10^5^ cells/well) in 100 μL of Waymouth’s medium. After 24 h of growth, the cells were treated with a series of neutral CPOH solutions (1, 2, and 4 mg/mL) alone or with *S. Enteritidis* at the multiplicity of infection (MOI)~10 for 1, 3, 6, 12, or 24 h to assess the effect of CPOH on *S. Enteritidis*-infected LMH cells. At the end of each treatment, the medium was removed, the cells were washed, and 10 μL of fresh CCK-8 solution was added, then the cells were incubated at 37 °C for 2 h. The absorbance at 450 nm was read in a microplate reader (Spark, Tecan, Switzerland). In addition, the absorbance at 630 nm was used as a reference wavelength because *Salmonella* caused turbidity. The relative cell viability was expressed as a relative percentage of the control (solvent-treated cells) according to the following formula:

Relative cell viability (%) = [(A_sample_ − A_blank_)/(A_control_ − A_blank_)] × 100.

### 2.6. Adhesion Assay

This test was conducted according to Mechesso et al. [24]. Briefly, LMH cells were seeded on 24-well plates (Corning, Inc., Corning, NY, USA) at a density of 3 × 10^5^ cells/mL in antibiotic free Waymouth’s medium and the cells were allowed to adhere to the plate for 24 h at 37 °C and 5% CO_2_. The cells (~80% confluency) were washed with prewarmed Waymouth’s medium and then infected with *S. Enteritidis* (MOI~10) for 90 min in the presence of CPOH (1 and 2 mg/mL). The medium was removed from the infected cells and washed 3 times with warm PBS to remove unattached bacteria. Then, the cells were lysed by 100 μL of 1% Triton X-100 (Invitrogen) and incubated for 10 min at room temperature, then 900 μL of LB medium was added. The suspensions were gently homogenized by repeated up-and-down pipetting, the suspensions were serially diluted (10-fold serial dilution) using LB broth, and 100 μL from 3 dilutions was plated (usually the 1:100; 1:1000, and 1:10,000 dilutions) on LB agar and incubated overnight at 37 °C. The colonies were counted on the plates and the number of colony forming units (CFU/mL) of the adhered bacteria was calculated.

### 2.7. Invasion Assay (Gentamicin Protection Assay)

As described previously in the adhesion assay, the cells were stimulated by *S. Enteritidis* (MOI~10) and simultaneously treated with CPOH (1 and 2 mg/mL) for 90 min. Next, the cells were washed three times with 1 × PBS and incubated for 60 min in the Waymouth’s medium supplemented with gentamicin (100 μg/mL). The cell lysis and total number (CFU/mL) of intracellular bacteria were determined following a similar procedure described previously in the adhesion assay.

### 2.8. RNA Isolation, cDNA Synthesis, and Real-Time Quantitative PCR

The LMH cells were first seeded on 6-well plates (Corning, Inc., Corning, NY, USA) (1 × 10^6^ cells/well) in 2000 μL of Waymouth’s medium supplemented with 10% FBS. After 24 h of growth, the cells were treated with *S. Enteritidis* (MOI~10) and two doses of neutral CPOH (1 and 2 mg/mL). At the end of each treatment, the medium was removed, and the total RNA was extracted using RNase-free lysis buffer containing 1% 2-mercaptoethanol using the PureLink RNA Mini Kit (Invitrogen). The lysate was homogenized and centrifuged at 2600× *g* for 5 min. The supernatant was mixed with chloroform (257 μL/mL) following the manufacturer’s recommendations and centrifuged at 12,000× *g* for 15 min at 4 °C to induce phase separation. The RNA in the supernatant was mixed with an equal volume of 70% ethanol and passed through the membrane cartridges. The samples were treated with a DNAase enzyme (Invitrogen), and after washing the RNA was eluted in RNase-free water. The RNA quantity was assessed using a NanoDrop Spectrophotometer (NanoDrop Technologies, Wilmington, DE, USA) by measuring the absorbance at 260 nm, and the RNA purity was determined using the optical density ratios at 260/280 and 260/230. The eluted RNA was stored at −80 °C. The total RNA (1 μg) was reverse transcribed to the complementary DNA following the kit’s instructions (Applied Biosystems, Beverly, MA, USA). The cDNA samples were stored at −80 °C. The expression levels of invasion-related genes (*Cdc-42*, *RhoG*, and *rac-1*) and antioxidant genes (nuclear factor erythroid 2-related factor 2 (*Nrf2*), heme oxygenase-1(*HO-1*), thioredoxin (*Txn*), and glutamate cysteine ligase catalytic subunit (*Gclc*)) in host cells were determined using a CFX Connect Real-Time PCR Detection System (Bio-Rad, Irvine, CA, USA) with samples containing 5 µL of SYBR Green Master Mix (Applied Biosystems, Beverly, MA, USA), 1 µL forward and reverse primer (Invitrogen) (Table 1), 1 µL cDNA template, and 2 µL RNA-free water. The reaction conditions involved one cycle at 95 °C for 2 min and 42 cycles of 95 °C for 15 s, 59 °C for 15 s, and 72 °C for 1 min. The expression experiments were performed three times with chicken *β-actin* as an internal standard. The relative gene expression levels were calculated using the 2^−(ΔΔCt)^ method [25].

### 2.9. Statistical Analysis

The data were analyzed using the factorial ANOVA (4 × 2 factorial design) and Tukey’s paired *t* test (*p* ≤ 0.05) procedures in SAS^®^ version 9.4 for Windows. Where significant differences were observed, Tukey’s test was used to compare the means. The final model was as follows:

*y_ijk_* = *μ* + *T_i_* + *S_j_* + *(TS)ij* + *eijk*

where *y_ijk_* represents the observations for dependent variables, *μ* is the overall mean of the experimental population, *T_i_* (*i* = 1 to 4) is the fixed effect of the treatment (CPOH), and *S_j_* (*j* = 1 to 2) is the random effect of *S. Enteritidis. TSij* is the interaction effect of the treatment and *Salmonella*, and *eijk* is the residual error.

## 3. Results

### 3.1. Antibacterial Effect of Neutral Cranberry Pomace (CPOH) on the Growth of S. Enteritidis

To evaluate whether CPOH had a direct antibacterial effect on *S. Enteritidis*, we used broth micro-dilution and spot assays. The MICs and MBCs values of the acidic CPOH were 16 and 32 mg/mL, respectively, against *S. Enteritidis*, while the neutral CPOH did not show any antibacterial activities against *S. Enteritidis* up to 64 mg/mL. It seems that the lower pH in acidic CPOH was responsible for the observed antimicrobial activities.

### 3.2. Effects of Neutral CPOH and S. Enteritidis on LMH Cell Viability

**Figure 1:**
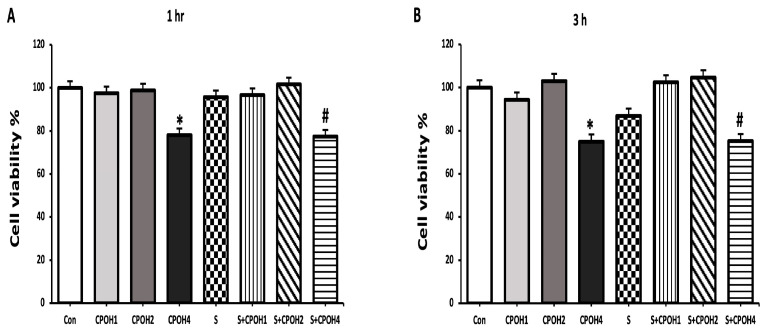

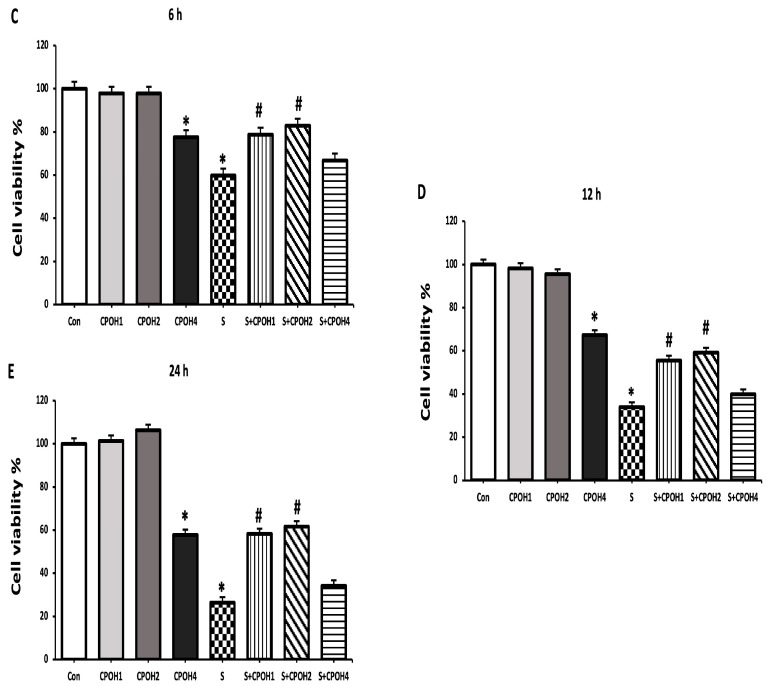
Protective effects of different doses of CPOH on LMH cell viability in the absence or presence of *Salmonella* at different time points: (**A**) 1 h; (**B**) 3 h; (**C**) 6 h; (**D**) 12 h; (**E**) 24 h. Control group (Con; LMH cells with growth medium), CPOH1 (LMH cells treated with 1 mg/mL neutral ethanolic extracted cranberry pomace), CPOH2 (LMH cells treated with 2 mg/mL neutral ethanolic extracted cranberry pomace), CPOH4 (LMH cells treated with 4 mg/mL neutral ethanolic extracted cranberry pomace), S (LMH cells infected with *S. Enteritidis*), S + CPOH1 (LMH cells simultaneously infected with *S. Enteritidis* and treated with 1 mg/mL neutral ethanolic extracted cranberry pomace), S + CPOH2 (LMH cells simultaneously infected with *S. Enteritidis* and treated with 2 mg/mL neutral ethanolic extracted cranberry pomace), and S + CPOH4 (LMH cells simultaneously infected with *S. Enteritidis* and treated with 4 mg/mL neutral ethanolic extracted cranberry pomace). Data are shown as average percentages of live cells, normalized to the Con at different time points. Data are representative of three independent experiments and shown as means ± SD; * *p* < 0.05 vs. the Con group; ^#^ *p* < 0.05 vs. the S group.

To investigate the potential effects of neutral CPOH on LMH cell viability when infected by *S. Enteritidis*, dose–response and time course experiments were carried out. LMH cells were treated with 0, 1, 2, or 4 mg/mL of neutral CPOH and simultaneously incubated with *S. Enteritidis* for 1, 3, 6, 12, or 24 h. Along the time course of the experiment, lower doses of neutral CPOH (1 and 2 mg/mL) did not affect the viability of the LMH cells but the high dose of CPOH 4 mg/mL significantly affected the viability of the LMH cells as compared with the Con group (Figure 1).

Starting after 6 h, the cell viability of the LMH cells in the S group was significantly lower than that in the Con group, while the cell viability in the S + CPOH1 and S + CPOH2 groups was markedly higher than that in the S group at 6, 12, and 24 h (*p* < 0.05; Figure 1C–E). These results indicated that low doses of neutral CPOH did not affect the LMH cells’ viability, while *S. Enteritidis*-induced cytotoxic effects after 6 h infection on LMH cells. Moreover, low doses of CPOH (1 and 2 mg/mL) alleviated the toxic effect of *S. Enteritidis* on LMH cells.

### 3.3. Effects of Neutral CPOH on S. Enteritidis Adhesion and Invasion to LMH Cells

**Figure 2:**
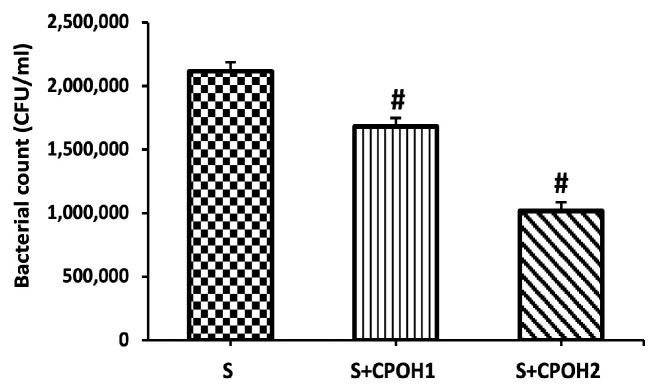
Effects of different doses of neutral CPOH on adhesion of *S. Enteritidis* to LMH cells: S (LMH cells infected with *S. Enteritidis* (MOI~10)), S + CPOH1 (LMH cells simultaneously infected with *S. Enteritidis* (MOI~10) and treated with 1 mg/mL neutral ethanolic extracted cranberry pomace), and S + CPOH2 (LMH cells simultaneously infected with *S. Enteritidis* (MOI~10) and treated with 2 mg/mL neutral ethanolic extracted cranberry pomace). Data are representative of three independent experiments and shown as means ± SD; ^#^ *p* < 0.05 vs. S group. CFU, colony-forming unit.

To test the ability of neutral CPOH on *S. Enteritidis* adhesion to LMH cells, the LMH cells were infected by *S. Enteritidis* (MOI~10) and simultaneously treated with neutral CPOH (1 and 2 mg/mL) for 90 min. Both doses (1 and 2 mg/mL) of neutral CPOH significantly reduced the bacterial adhesion to the LMH cells (*p* < 0.05; Figure 2). The results showed that neutral the CPOH had antiadhesion activity against *S. Enteritidis* to LMH cells.

**Figure 3:**
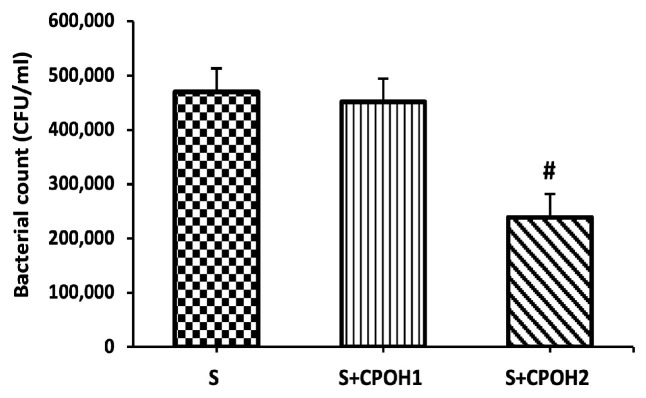
Effects of different doses of neutral CPOH on invasion of *S. Enteritidis* into LMH cells: S (LMH cells infected with *S. Enteritidis* (MOI~10)), S + CPOH1 (LMH cells simultaneously infected with *S. Enteritidis* (MOI~10) and treated with 1 mg/mL neutral ethanolic extracted cranberry pomace), and S + CPOH2 (LMH cells simultaneously infected with *S. Enteritidis* (MOI~10) and treated with 2 mg/mL neutral ethanolic extracted cranberry pomace). Data are representative of three independent experiments and shown as means ± SD; ^#^ *p* < 0.05 vs. the S group. CFU, colony-forming unit.

In order to investigate whether the antiadhesion activity of neutral CPOH could inhibit *S. Enteritidis* invasion into LMH cells, a gentamicin assay was applied. Only 2 mg/mL of neutral CPOH reduced the bacterial invasion into the LMH cells, while 1 mg/mL of neutral CPOH did not protect the LMH cells from *S. Enteritidis* invasion (*p* < 0.05; Figure 3). These results showed that neutral CPOH had the potential to protect poultry liver from *S. Enteritidis* adhesion to and invasion into LMH cells.

### 3.4. Impacts of Neutral CPOH on Expression of Invasion-Related Host Cell Protein Genes

**Figure 4:**
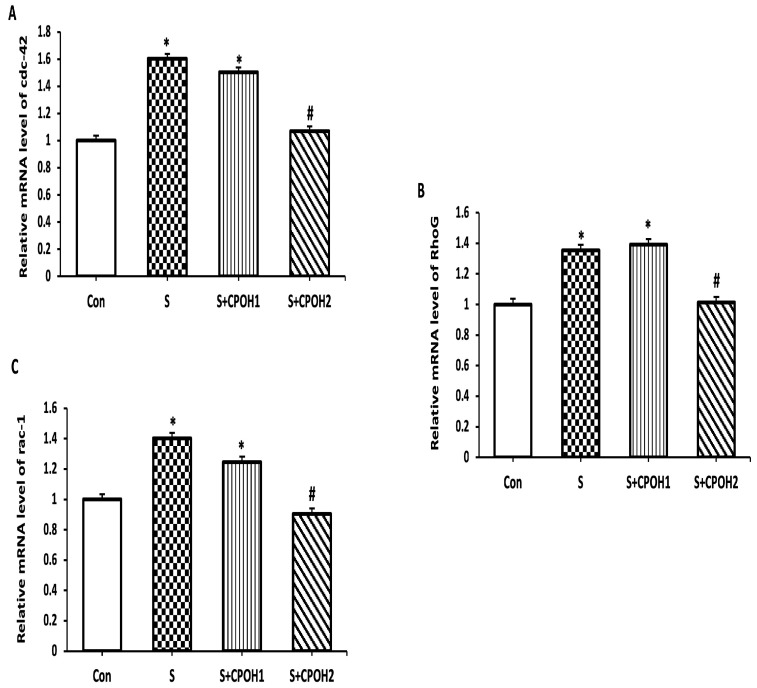
Effects of different doses of neutral CPOH on expression of invasion-related host cell proteins. The gene expression levels of *cdc-42* (**A**), *RhoG* (**B**), and *rac-1* (**C**) were determined in LMH cells treated with various concentrations of neutral CPOH (mg/mL): control group (Con; LMH cells with growth medium), S (LMH cells infected with *S. Enteritidis)*, S + CPOH1 (LMH cells simultaneously infected with *S. Enteritidis* and treated with 1 mg/mL neutral ethanolic extracted cranberry pomace), and S + CPOH2 (LMH cells simultaneously infected with *S. Enteritidis* and treated with 2 mg/mL neutral ethanolic extracted cranberry pomace). Gene expression was determined using RT-qPCR and is represented relative to *b-actin*. Relative gene expression levels were calculated using the 2^−(ΔΔct)^ method. Data are representative of three independent experiments and shown as means ± SD; * *p* < 0.05 vs. Con group; ^#^ *p* < 0.05 vs. S group.

To confirm the effect of neutral CPOH against *S. Enteritidis* invasion, the expression of invasion-related host cell proteins was evaluated in LMH cells. The LMH cells treated by *S. Enteritidis* alone significantly increased the expression of *cdc-42*, *RhoG,* and *rac-1* in comparison with the Con group. CPOH1 did not affect the expression of *cdc-42*, *RhoG,* or *rac-1* compared to the S group, while the treatment of cells with CPOH2 downregulated the expression levels of *cdc-42, RhoG,* and *rac-1*(*p* < 0.05; Figure 4A–C). These results indicate that neutral CPOH inhibited *S. Enteritidis* invasion into poultry liver cells by downregulating the expression of invasion-related host cell proteins (*cdc-42, RhoG,* and *rac-1*).

### 3.5. Effects of Neutral CPOH on Expression of Antioxidant-Related Genes in LMH Cells

**Figure 5:**
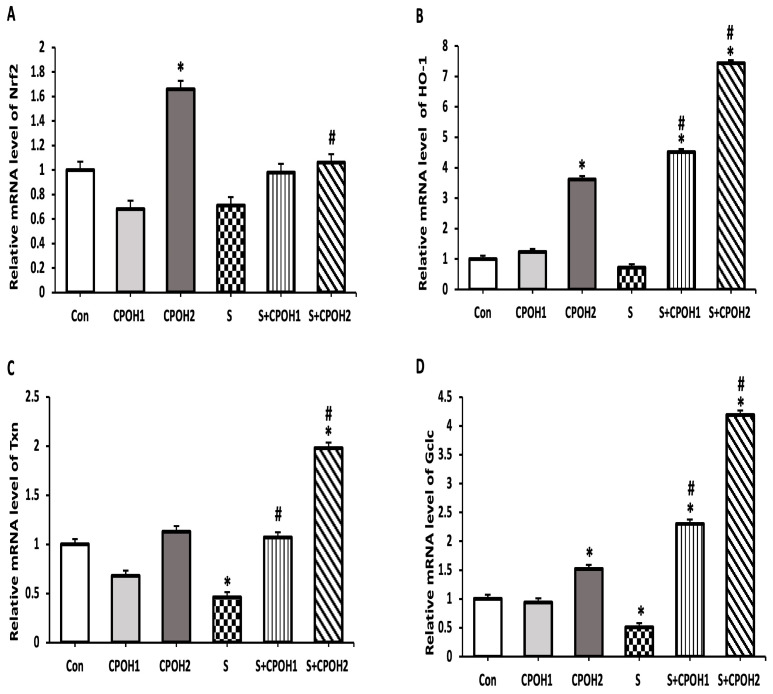
Effects of different doses of neutral cranberry pomace after 6 h of treatment on expression of 4 antioxidant-related genes, including *Nrf2* (**A**), *HO-1* (**B**), *Txn* (**C**), and *Gclc* (**D**). Control group (LMH cells with growth medium), CPOH1 (LMH cells treated with 1 mg/mL neutral ethanolic extracted cranberry pomace), CPOH2 (LMH cells treated with 2 mg/mL neutral ethanolic extracted cranberry pomace), S (LMH cells infected with *S. Enteritidis*), S + CPOH1 (LMH cells simultaneously infected with *S. Enteritidis* and treated with 1 mg/mL neutral ethanolic extracted cranberry pomace), and S + CPOH2 (LMH cells simultaneously infected with *S. Enteritidis* and treated with 2 mg/mL neutral ethanolic extracted cranberry pomace). Gene expression was determined using RT-qPCR and is represented relative to *b-actin.* Relative gene expression levels were calculated using the 2^-(∆∆Ct)^ method. Data are representative of three independent experiments and shown as means ± SD; * *p* < 0.05 vs. Con group; ^#^ *p* < 0.05 vs. S group.

**Figure 6:**
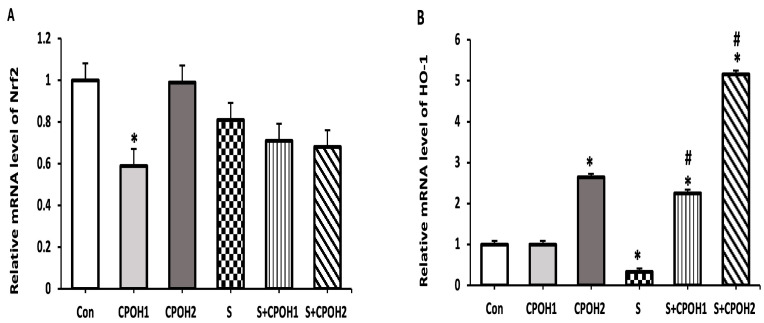

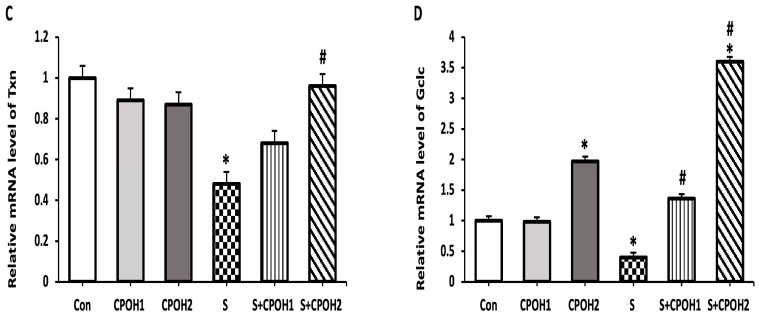
Effects of different doses of neutral cranberry pomace after 12 h of treatment on the expression of 4 antioxidant-related genes, including *Nrf2* (**A**), *HO-1* (**B**), *Txn* (**C**), and *Gclc* (**D**). Control group (LMH cells with growth medium), CPOH1 (LMH cells treated with 1 mg/mL neutral ethanolic extracted cranberry pomace), CPOH2 (LMH cells treated with 2 mg/mL neutral ethanolic extracted cranberry pomace), S (LMH cells infected with *S. Enteritidis*), S + CPOH1 (LMH cells simultaneously infected with *S. Enteritidis* and treated with 1 mg/mL neutral ethanolic extracted cranberry pomace), and S + CPOH2 (LMH cells simultaneously infected with *S. Enteritidis* and treated with 2 mg/mL neutral ethanolic extracted cranberry pomace). Gene expression was determined using RT-qPCR and is represented relative to *b-actin.* Relative gene expression levels were calculated using the 2^-(∆∆Ct)^ method. Data are representative of three independent experiments and shown as means ± SD; * *p* < 0.05 vs. Con group; ^#^ *p* < 0.05 vs. S group.

In order to determine other possible cyto-protective mechanisms of neutral CPOH on LMH cells infected by *S. Enteritidis*, the expression levels of four antioxidant-related genes (*Nrf2*, *HO-1*, *Txn,* and *Gclc,*) were measured at 6 and 12 h. The results at 6 h are shown in Figure 5. The expression levels of *Txn* and *Gclc* mRNA were significantly decreased in the S groups as compared to the Con group. The expression levels of *HO-1, Txn,* and *Gclc* mRNAs, but not *Nrf2,* were significantly elevated in the S + CPOH1 group compared to S group, while the expression levels of all four genes, *Nrf2, HO-1, Txn,* and *Gclc,* were upregulated in the S + CPOH2 group in comparison with the S group. Similarly, at 12 h (Figure 6), the *HO-1*, *Txn,* and *Gclc* gene expression levels were significantly downregulated in the S group as compared to the Con group. The gene expression levels of both *HO-1* and *Gclc* genes were elevated in the S + CPOH1 group compared to the S group at 12 h, while the expression levels of *HO-1, Txn,* and *Gclc* were induced in the S + CPOH2 group as compared to the S group. These results suggest that the neutral CPOH upregulated the expression of antioxidant genes against *S. Enteritidis*-induced oxidative stress, consequently protecting chicken liver cells from *S. Enteritidis*-induced damage.

## 4. Discussion

*S. Enteritidis* is a globally significant zoonotic foodborne pathogen leading to large numbers of deaths in humans and causing economic losses to the poultry industry. Virulent *S. Enteritidis* strains can colonize in the intestine and spread systemically throughout the chicken, causing a persistent infection of the poultry liver. *Salmonella enterica* serovar Typhimurium-infected poultry show prominent liver lesions, with numerous pale necrotic foci that are infiltrated by numerous heterophilic cells, in addition to pyknosis of liver nuclei [26]. LMH cells (ATCC^®^ CRL-2117™) have been previously used as a model for *Salmonella enterica* infection in poultry [27,28,29]. Even though the complete picture of the pathogenesis of *Salmonella* infection is still not very clear, it mainly contains three steps: (i) attachment and adherence to the surface of the host cell and secretion of effector proteins, allowing host cell invasion; (ii) initial multiplication; (iii) overcoming host defense mechanisms [30]. Effective methods of elimination and eradication of intracellular *S. Enteritidis* are still very limited because of the ability of this pathogen to survive, spread, and persist within the host cells. In addition, the emergence of multi-antimicrobial-resistant *Salmonella* strains from poultry production has added additional challenges for controlling Salmonellosis. These issues highlight the need for the identification of innovative, cost-effective, and environmentally friendly antibiotic alternatives that are capable of reducing health hazards and economic losses due to *S. Enteritidis*. The use of phytochemicals represents one of many potential solutions being investigated, since they contain many bioactive compounds with potent antimicrobial activities [31].

Cranberry pomace can be an excellent sustainable source of inexpensive natural bioactive compounds as an immune modulator, antioxidant, and antimicrobial agent. In particular, cranberry pomace exhibited a positive effect on broiler immunity by reducing the expression of the inflammation-related gene IL-4 (Interleukin-4) in the liver [32]. CPOH contains high levels of total phenolics, tartaric esters, flavanols, and anthocyanins, which increase the antioxidant and antimicrobial efficacy of the pomace. Notably, the phenolic acids, tartaric esters, and antioxidant activity of CPOH were 3–4 times higher than those of the organic cranberry pomace (CP) itself, while the flavanols and anthocyanins were 5 and 6 times higher, respectively [16]. It is possible that the phytochemicals included in cranberry products have a variety of pleiotropic mechanisms of action against bacteria. Furthermore, the synergistic antibacterial actions among the bioactive agents may perform a broad range of antimicrobial activities [33]. In addition, CPOH could improve the effectiveness of a wide range of antibiotics, so its capacity to potentiate the action of antibiotics could hinder the emergence of antibiotic-resistant infections [34]. Interestingly, cranberry can prevent many of the enteric infections in the body and may offer a potential alternative to antibiotics [35,36]. Therefore, this study attempted to extend our previous findings [16,18,32,33] and explore the protective effects of neutral CPOH on chicken hepatic cells during *S. Enteritidis* infection.

Firstly, we studied the bactericidal effect of neutral CPOH directly on *S. Enteritidis* growth. Our results showed that neutral CPOH up to 64 mg/mL did not affect the growth of *S. Enteritidis.* Thus, the antibacterial effect might be relevant to other mechanisms.

In order to select biologically safe concentrations of CPOH, we tested the effects of different doses of CPOH on LMH cells infected with *S. Enteritidis* and measured the cell viability at different time points. The LMH cells treated with different doses of acidic CPOH showed extreme cytotoxic effects (data not shown). Accordingly, we neutralized the CPOH first. Interestingly, our data showed for the first time that the neutral CPOH up to 2 mg/mL had no effect on the viability of the chicken liver cells throughout the 24 h of treatment, while the higher dose of neutral CPOH (4 mg/mL) decreased the percent of viable cells. In accordance with our findings, a previous study reported that the highly acidic nature of the cranberry juice adversely affected the viability of the human oral epithelial cells, while the de-acidified cranberry juice improved the viability of the oral epithelial cells [37]. Additionally, Harmidy et al. [35] showed that cranberry proanthocyanin concentrations ranging from 0–100 µg/mL had no significant change in HeLa cell viability, while the higher concentration of 200 µg/mL showed a slight increase in the number of dead cells.

Our study revealed that *S. Enteritidis* infection decreased chicken liver cell viability starting from 6 h. Another study by Chu et al. [38] reported that *S.* Infantis induced a significant reduction in the cell survival rate of human intestinal cells at 6 h post-infection and remained almost unchanged after 8 h post-infection. More importantly, our results revealed the excellent potential of neutral CPOH to combat *S. Enteritidis*-induced hepatic epithelial cell damage, since both 1 and 2 mg/mL of neutral CPOH significantly increased the percentages of LMH cell viability at 6, 12, and 24 h post-*S. Enteritidis* infection. Consistent with our result, Xiong et al. [39] reported a restoration in the viability of Caco-2 cells infected with lipopolysaccharides (LPS) following icariin (another flavonoid plant extract) treatment.

To explore the underlying mechanisms by which neutral CPOH alleviated *S. Enteritidis*-induced hepatic epithelial cell damage, we first evaluated the effect of neutral CPOH on *S. Enteritidis* adhesion to and invasion into LMH cells. The prevention of bacterial adhesion is a critical step to interfere with bacterial pathogenesis and colonization at the early phase of infection [40]. Flavanones are well-known for their antiadhesive properties. Flavanones in citrus pomace extract have antiadhesive properties, decreasing the adhesion of *S*. Typhimurium to Caco-2 cells by 20–75% [41]. Although several previous studies revealed that the anti-adherence properties of cranberry against *E. coli* especially in renal epithelial cells, limited studies have focused on the anti-adherence activity of cranberry against other Gram-negative bacteria, in particular *Salmonella* species in chicken hepatic cells [42,43]. In fact, Das et al. [18] showed that the treatment of *Salmonella* with acidic CPOH in the bacterial culture setting led to the downregulation of a set of *Salmonella* virulence genes (effector proteins), including those associated with the motility, adherence, and invasion of host cells by *S. Enteritidis*. In addition, Lau et al. 2019 [44] showed the antimicrobial effect of CPOH on different foodborne pathogens in different bacterial growth media. However, these previous studies did not investigate the antiadhesive and anti-invasive effects of CPOH on the chicken cells infected by *S. Enteritidis*, nor did they study the underling mechanism from the host cell angle. Hence, in the present study, we were concerned about the efficacy of CPOH in modulating the host–pathogen interaction process. Interestingly, our data showed that neutral CPOH decreased *S. Enteritidis* adherence to chicken hepatic epithelial cells. This could be due to reducing the formation and expression of bacterial adhesins (the release or formation of defective adhesins) or by interfering with the functionality of adhesins (binding) by some cranberry bioactive compounds [33]. In fact, Das et al. [18] suggested that the treatment of *Salmonella* with acidic CPOH in broth led to the downregulation of a set of *Salmonella* virulence genes (effector proteins), including those associated with the motility, adherence, and invasion of host cells by *S. Enteritidis*. In the present study, we found that the neutral CPOH cells (2 mg/mL) have the ability to inhibit *S. Enteritidis* invasion into chicken hepatic cells. *Salmonella* may invade the cultured epithelial cells by injecting different effector proteins into the host cells, which in turn activate the host cell Rho GTPases such as Cdc-42, RhoG, and rac-1 and finally induce the rearrangement of the host cell actin cytoskeleton, membrane ruffling, and internalization [45]. To corroborate this assumption, we measured the expression of the invasion-related host genes, and our results showed that neutral CPOH significantly downregulated the expression of *Cdc-42*, *RhoG,* and *rac-1* genes in *S. Enteritidis*-infected LMH cells as compared to the S group. Similar inhibition of *rac-1* expression was previously observed by Kabirifar et al. [46], who reported that quercetin, one of abundant flavonoids in berry fruits, protected the liver from the injury induced by bile duct ligation in rats.

Another possible protective effect of neutral CPOH on LMH cells against *S. Enteritidis*-induced damage was the expression of many genes related to the antioxidant defense system. The upregulation of antioxidant genes is a cellular adaptation to oxidative stress and is mainly controlled by the Nrf2 pathway [47]. Nrf2 is a transcription factor that coordinates a massive array of cytoprotective genes including glutathione (Gclc) and thioredoxin (Txn)-based antioxidant systems, NADPH regeneration, and heme and iron metabolism (heme oxygenase 1(HO-1)) [10]. Another key finding of this study is it showed that *S. Enteritidis* significantly downregulated the expression of *HO-1*, *Txn,* and *Gclc* genes, while neutral CPOH countered these *S. Enteritidis*-induced effects. Onyiah et al. [48] reported that the treatment of *S.* Typhimurium-infected intestinal epithelial cells with synthetic compounds to promote *HO-1* gene expression led to the inhibition of inflammatory cytokine expression and could protect the intestinal epithelial cells from inflammatory damage. Furthermore, decreased bacterial colonization upon the administration of antioxidant reagents was seen in *Salmonella* infections [49]. Our study revealed that neutral CPOH induced the expression of *Nrf2*, *HO-1*, *Txn,* and *Gclc* genes in *S. Enteritidis*-infected chicken hepatic cells. Thus, our findings contribute new and valuable evidence for the possible antioxidant mechanism of CPOH and its protection of chicken liver cells against *S. Enteritidis*-induced damage.

## 5. Conclusions

Based on our results, we concluded that *S. Enteritidis* colonized and damaged chicken hepatic cells by adhering to and invading into LMH cells, as well as by downregulating the antioxidant genes in LMH cells, whereas CPOH protected the chicken hepatic cells against these adverse effects induced by *S. Enteritidis*. The anti-infective properties of neutral CPOH noted here may be associated with its inhibitory effect on *S. Enteritidis* adhesion to and invasion into chicken hepatic cells, as well as by increasing the expression of *Ho-1*, *Gclc,* and *Txn* antioxidant genes. Obviously, the possibility of the existence of other mechanisms cannot be eliminated. Nevertheless, our results suggest that CPOH can be used as a promising non-antibiotic alternative against *S. Enteritidis* infection in chicken liver cells.

## Figures and Tables

**Table 1:** Primers used in the study for the quantitative real-time PCR testing.

Gene	Primer Sequence	
*cdc-42*	5-TGGTGGTGCATCTCCCTATG-3 5-ATGGTGCCATGCTGAACACT-3	Forward Reverse
*RhoG*	5-TGCAGAGCATCAAATGCGTG-3 5-GGCGATGGAGAAGCAGATGA-3	Forward Reverse
*rac-1*	5-ACCCCCAAACAGATGTCTTCTTA-3 5-TGCAACCAAGCCCTTACCAA-3	Forward Reverse
*Nrf2*	5-CTGCTA GTG GATGGCGAGAC-3 5-CTC CGA GTT CTC CCC GAA AG-3	Forward Reverse
*HO-1*	5-AGCTTCGCACAAGGAGTG TT-3 5-GGAGAGGTGGTCAGCATG TC-3	Forward Reverse
*Txn*	5-GTGCATGCCAACATTCCA GT-3 5-CTCCATGGCGGGAGATTAGAC-3	Forward Reverse
*Gclc*	5-GGA CGC TAT GGG GTT TGG AA-3 5-AGG CCA TCA CAA TGG GAC AG-3	Forward Reverse
*β-actin*	5-ATCTTTCTTGGGTATGGAGTC-3 5-GCCAGGGTACATTGTGG-3	Forward Reverse

## Data Availability

All data presented in this research are available through the corresponding author.
